# Nocebo effects in visceral pain: concept and design of the experimental randomized-controlled pain study ‘NoVis’

**DOI:** 10.3389/fpsyt.2023.1270189

**Published:** 2023-10-13

**Authors:** Jana Luisa Aulenkamp, Adriane Icenhour, Sigrid Elsenbruch

**Affiliations:** ^1^Department of Anesthesiology and Intensive Care Medicine, University Hospital Essen, University of Duisburg-Essen, Essen, Germany; ^2^Department of Neurology, Center for Translational Neuro- and Behavioral Sciences (C-TNBS), University Hospital Essen, University of Duisburg-Essen, Essen, Germany; ^3^Department of Medical Psychology and Medical Sociology, Ruhr University Bochum, Bochum, Germany

**Keywords:** gut-brain axis, nocebo effect, visceral pain, somatic pain, visceral hyperalgesia, treatment expectations, stress, fear

## Abstract

The role of psychological factors in the pathophysiology and treatment of chronic visceral pain in disorders of gut-brain interactions (DGBI) is increasingly appreciated. Placebo research has underscored that expectations arising from the psychosocial treatment context and from prior experiences shape treatment responses. However, effects of negative expectations, i.e., nocebo effects, as they are likely crucial elements of DGBI patients’ clinical reality, have thus far only rarely been investigated in the context of visceral pain, with untapped potential for improved prevention and treatment. The experimental randomized-controlled pain study “NoVis,” carried out within the Collaborative Research Center (CRC) 289 (“Treatment Expectation”), aims to close gaps regarding the generation and persistence of nocebo effects in healthy volunteers. It is designed to elucidate effects of negative expectations in a multiple-threat paradigm with intensity-matched rectal distensions and cutaneous thermal stimuli, allowing to test nocebo effects in the visceral and somatic pain modalities. Negative expectations are experimentally induced by elements of doctor-patient communication (i.e., instruction) and/or by surreptitious amplification of symptom intensity (i.e., experience/learning) within a treatment context. Accordingly, the repeated measures between-subject design contains the between-group factors “treatment instruction” (negative vs. control) and “treatment experience” (negative vs. control), with volunteers randomized into four experimental groups undergoing several pain stimulation phases (repeated factor). This allows to compare the efficacy of instruction vs. experience, and more importantly, their combined effects on the magnitude of negative expectations and their impact on pain responses, which we expect will be greatest for the visceral modality. After a *Baseline*, short-term effects are assessed during a test phase accomplished on study day 1 (*Test-1 Phase*). To explore the persistence of effects, a second test phase is accomplished 1 week later (*Test-2 Phase*). Effects of negative expectations within and across pain modalities are assessed at the subjective and objective levels, with a focus on psychophysiological and neuroendocrine measures related to stress, fear, and anxiety. Since nocebo effects can play a considerable role in the generation, maintenance, or worsening of chronic visceral pain, and may even constitute risk factors for treatment failure, knowledge from experimental nocebo research has potential to improve treatment outcomes in DGBI and other clinical conditions associated with chronic visceral pain.

## 1. Introduction

Chronic visceral pain is an important yet often underestimated clinical and societal problem not only in European countries like Germany (1) but worldwide (2, 3). From a clinical perspective, chronic or recurring pain arising from the viscera is highly prevalent, causes enormous suffering and significant healthcare expenditures (4). Effective treatment is notoriously difficult, especially in the absence of identifiable organic pathology, as is characteristic of functional gastrointestinal disorders such as the irritable bowel syndrome (IBS) and functional dyspepsia (FD) (5, 6). Since these conditions demonstrably involve alterations in gut-brain signaling, they are now based on international expert consensus referred to as disorders of gut-brain interactions (DGBI) (7). Treatment challenges are owed at least in part to the multifactorial etiology of DGBI involving biological, psychological, and social factors, embedded within a biopsychosocial model (8). Although the pathophysiology remains incompletely understood, it is assumed to involve visceral hypersensitivity and interoceptive hypervigilance, resulting from disturbances of the microbiome-gut-brain axis. Hence, from a mechanistic perspective, the complexity of the microbiome-gut-brain axis with its multitude of afferent and efferent pathways that connect the enteric nervous system and microbiome of the gastrointestinal (GI) tract with the central nervous system (CNS) poses research challenges to be resolved as a basis for improved treatment. One research target with great potential for successful translation into patient care is the brain as the organ where visceral signals are perceived as painful yet can also be modulated by psychological factors that can inform behavioral treatment approaches.

Psychological factors, including cognitions and emotions, modulate GI sensorimotor functions and illness behaviors, and thereby fundamentally contribute to the etiology, pathophysiology, and treatment of chronic visceral pain in DGBI. This is exemplified by long-standing research on the role of stress and stress mediators in visceral hyperalgesia (5, 9, 10), as well as by more recent work into interoceptive fear underlying hypervigilance and maladaptive avoidance (11). These psychological research lines have advanced mechanistic knowledge and paved the way toward translation into clinical application. Indeed, cognitive-behavioral treatment concepts targeting visceral pain are increasingly appreciated, continuously refined and more broadly disseminated (12, 13). Despite these impressive examples for successful translation from mechanistic studies in animals and humans into patient care, the potential of psychological concepts together with research tools from the cognitive and affective neurosciences is far from exhausted. At the interface of neurogastroenterology, the pain field and the cognitive neurosciences, transdisciplinary research into placebo and nocebo effects in visceral pain has great potential to advance mechanistic knowledge and improve treatment options for patients with chronic visceral pain (1, 5).

Placebo and nocebo effects demonstrably shape treatment outcomes, including patient-reported pain. They are mediated by treatment expectations arising from prior treatment experience as well as from communication and information provided by healthcare professionals, the media, or peers within any given psychosocial treatment context. Existing knowledge regarding treatment expectancy effects in chronic visceral pain comes primarily from studies conducted in the context of DGBI (1) as well as of chronic-inflammatory bowel diseases (14). High placebo response rates in clinical trials have initially drawn rather negative attention as a “nuisance” hampering successful drug development for treatment of pain-related gastrointestinal conditions (1, 15). Yet, it was also in patients with IBS that early brain imaging studies on the neural mechanisms underlying visceral placebo analgesia have been accomplished (16–18), paving the way for mechanistic work in other pain models and chronic pain conditions. In parallel to evolvement of research efforts and tools to study the placebo effect, treatment approaches built on positive treatment expectations (“placebo interventions”) have been successfully employed in IBS (19–21), inspiring efforts to utilize positive expectancy effects in the treatment of patients with other acute or chronic pain conditions [e.g., (22–25)]; and to further conceptual work on their role in psychotherapy (26).

Nocebo effects, mediated by negative expectations, remain less well-understood, although their scientific and clinical relevance undoubtedly rivals that of the placebo effect. In patients with DGBI, negative expectations and treatment-related worries and fears must be viewed as a part of the clinical treatment reality. The experience of sudden symptom aggravation as part of the typical waxing and waning of symptoms, worries about treatment choices and their possible side effects, and even repeated treatment failures are examples of how negative expectations could be generated and maintained in patients’ clinical reality. Such experiences shape cognitive and emotional responses, increasing maladaptive coping, catastrophizing, GI-specific fear and anxiety, and chronic stress burden. Together, these factors can contribute to visceral hyperalgesia and interoceptive hypervigilance as key mechanisms underlying the transition from acute to chronic visceral pain in patients. Hence, nocebo effects may play a considerable role in the generation, maintenance, or worsening of chronic gastrointestinal symptoms, and could even constitute risk factors for treatment failure (27). Given broad implications for the overlapping fields of gastroenterology, psychosomatic medicine, and pain (1, 28), elucidating nocebo effects in visceral pain in laboratory settings is necessary as a basis for enhancing knowledge about underlying psychological and neurobiological mechanisms. Given evidence supporting distinct differences between visceral and somatic pain in terms of perceptual and emotional responses as well as to neural processing (29–31), dedicated research in clinically relevant visceral pain models is needed in order to complement and extend nocebo knowledge from the somatic pain field.

Building on long-standing knowledge on the role of nocebo mechanisms in the generation and worsening of nausea [reviewed in (1)], our group started experimental work to systematically study both placebo and nocebo effects in visceral pain and fecal urgency induced by rectal distension [reviewed in (27)]. Together, data from behavioral and neuroimaging studies in healthy volunteers as well as in patients with IBS and IBD support that treatment expectations effectively change the subjective response to objectively identical painful visceral stimuli in healthy individuals, inducing placebo analgesia or nocebo hyperalgesia, along with changes in neural activation in key brain regions (32–34) known to be involved in the processing of visceral pain (35). While this research focused primarily on positive treatment expectations underlying placebo effects, we also initiated work elucidating the effects of negative treatment expectations, employing negative treatment suggestions and (thus far to a much smaller extent) learning/conditioning procedures to induce negative expectations underlying nocebo effects. Providing specific written and verbal treatment information or creating distinct treatment experiences through learning/conditioning procedures constitute the two paradigms which have been implemented in order to experimentally generate and explore both placebo and nocebo effects in laboratory settings (36, 37). The distinction between “instruction” and “experience/conditioning” as principle psychological mechanisms has been the basis for different experimental study designs, although it is becoming increasingly clear that these processes are conceptually and clinically intertwined (27). Clearly, prior experience and learning processes shape and interact with treatment instruction within any given clinical or experimental context, giving rise to expectancies (38). The combination of instruction and experience has been shown to be particularly effective in eliciting a nocebo effect (39), but this has never been tested in the context of visceral pain. The putative relevance of stress-related state factors like anxiety, fear, and stress also remains unclear in the context of nocebo effects but is strongly supported by our study showing that acute psychosocial stress amplified visceral nocebo effects in healthy individuals (40). Given that visceral pain sensitivity is demonstrably more responsive to the acute stress mediator cortisol (31) and uniquely driven by pain-related fear (29) it is intriguing to speculate that nocebo effects may be more pronounced for sensations arising from the visceral modality, as interoceptive sensations, when compared to exteroceptive sensations like thermal cutaneous pain. The notion of more “powerful” visceral pain modulation by mechanisms engaged during nocebo responses is also of interest given altered visceral pain-related fear learning in IBS (28) as well as greater emotional and hypothalamic–pituitary–adrenal (HPA) axis stress responsivity in patients with IBS and IBD (10). Therefore, elucidating the role of fear, anxiety, and stress in the context of nocebo research is a timely and important goal of our experimental study concept and design.

We designed an experimental randomized-controlled study dedicated to elucidating the effects of negative treatment expectations on subjective and objective responses to acute pain stimuli in healthy volunteers. The design allows to test the effects of negative expectations, generated by negative treatment instructions as well as by negative treatment experience separately, and more importantly, allows to elucidate their putative interaction. Within a “multiple-threat paradigm,” visceral pain is induced by pressure-controlled rectal distensions, delivered as part of a pain stimulation series also containing intensity-matched thermal cutaneous stimuli (41), modeling symptom burden in patients with multiple comorbidities and allowing us to elucidate specificity to pain modality. Effects of negative expectations on pain intensity and pain unpleasantness are assessed as primary outcomes. As secondary outcomes, different measures related to fear and stress are measured using behavioral, neuroendocrine, and psychophysiological responses. To elucidate the persistence of nocebo effects over time, participants are re-exposed to pain stimuli 1 week later.

The specific aims are as follows:

*Aim 1*: To test dynamic changes in treatment- and pain-related expectations within and outside of the psychosocial treatment context, i.e., on study days 1 and 2, as mediators of nocebo effects.

*Aim 2*: To test the effects of negative treatment expectations induced by instruction and/or experience on subjective and objective responses to acute visceral and somatic pain stimuli.

*Aim 3*: To determine if a negative treatment experience enhances the magnitude of negative instruction effects.

*Aim 4*: To compare the magnitude of nocebo effects in the visceral versus the somatic pain modality.

*Aim 5*: To assess the persistence of nocebo effects on responses evoked by a re-exposure to pain stimuli 1 week later.

*Aim 6*: To identify predictors of interindividual variability in the magnitude of nocebo effects and to elucidate associations with state and trait variables relevant to stress, anxiety, and fear at the behavioral, psychophysiological, and neuroendocrine levels.

## 2. Methods

### 2.1. Setting, recruitment, and sample

This ongoing study is conducted at the University Hospital Essen, University of Duisburg-Essen, Germany, as part of the Collaborative Research Center (CRC) 289 “Treatment Expectation”, funded by the German Research Foundation (Deutsche Forschungsgemeinschaft, DFG). The overall goal of the CRC is to elucidate mechanisms and clinical implications of treatment expectations.^1^ Within the ongoing first funding phase (7/2020–6/2024), research goals are primarily focused on health outcomes relevant to patients with chronic pain and depression. The present study is accomplished as part of subproject A04 (PI: author S.E.), which addresses expectancy effects on pain with a unique focus on visceral pain and conditions involving the gut-brain axis. Ethical approval for research within subproject A04, including the present experimental study, was obtained from the ethics committee at University Hospital Essen (19-8897-BO). The study has been registered in the German Registry for Clinical Studies (DRKS: DRKS00024410). All participants sign consent (for details on two versions of consent forms, see section 2.4.3), and receive a financial compensation for their participation, comprising the two study days involving pain testing described herein, as well as additional assessments accomplished as part of the CRC’s central projects (see section 2.5.7).

The recruitment goal is to include a total of *N* = 120 healthy participants in the study. The sample size is based on *a priori* power analysis with G*Power (42) for interaction effects in a repeated measure ANOVA with the factors time and group. Assuming an effect size of *d* = 0.4 for interaction effects, *α* = 0.05, and 1-β = 0.90, the number of volunteers is *N* = 96. To account for possible exclusions or drop-out and to allow stratified recruitment of male and female volunteers, a group size of *N* = 30 subjects per final experimental group is intended.

To this end, healthy men and women between 18 and 45 years of age and a body mass index between 18 and 30 are recruited for a study on visceral vs. somatic pain perception by public advertisement in the surrounding universities, clinics, and community of the Ruhr area. The standardized recruitment and screening process consists of a structured telephone screening and a personal interview. Exclusion criteria include any acute or chronic health condition, with particular attention to recurring pain and frequent gastrointestinal symptoms based on self-report and an established questionnaire assessing various symptoms over the past 3 months (43), regular use of medications (except contraceptives and thyroid medication), elevated anxiety and depression scores based on the Hospital Anxiety and Depression Scale (HADS) (44)(subscale scores ≥8 used as cut-offs), pregnancy or breastfeeding (urinary pregnancy test on the first day of the study), and insufficient proficiency in the German language. A physical examination including a digital rectal examination is performed to exclude external or internal anorectal tissue damage which may interfere with rectal balloon placement. Given standardized brain scanning protocols accomplished as part of a CRC central project, the standard magnetic resonance imaging (MRI)-related exclusion criteria apply (e.g., metal implants, pacemaker, large tattoo in head/neck position, and known claustrophobia), and structural brain abnormalities are excluded in collaboration with the Institute of Diagnostic and Interventional Radiology and Neuroradiology. Given the study aims, especially the putative role of prior experiences with the experimental paradigms, any participation in an experimental pain study involving somatic or visceral pain stimuli within the previous 3 months (based on self-report) is also exclusionary.

### 2.2. Design and outcomes

**Figure 1:**
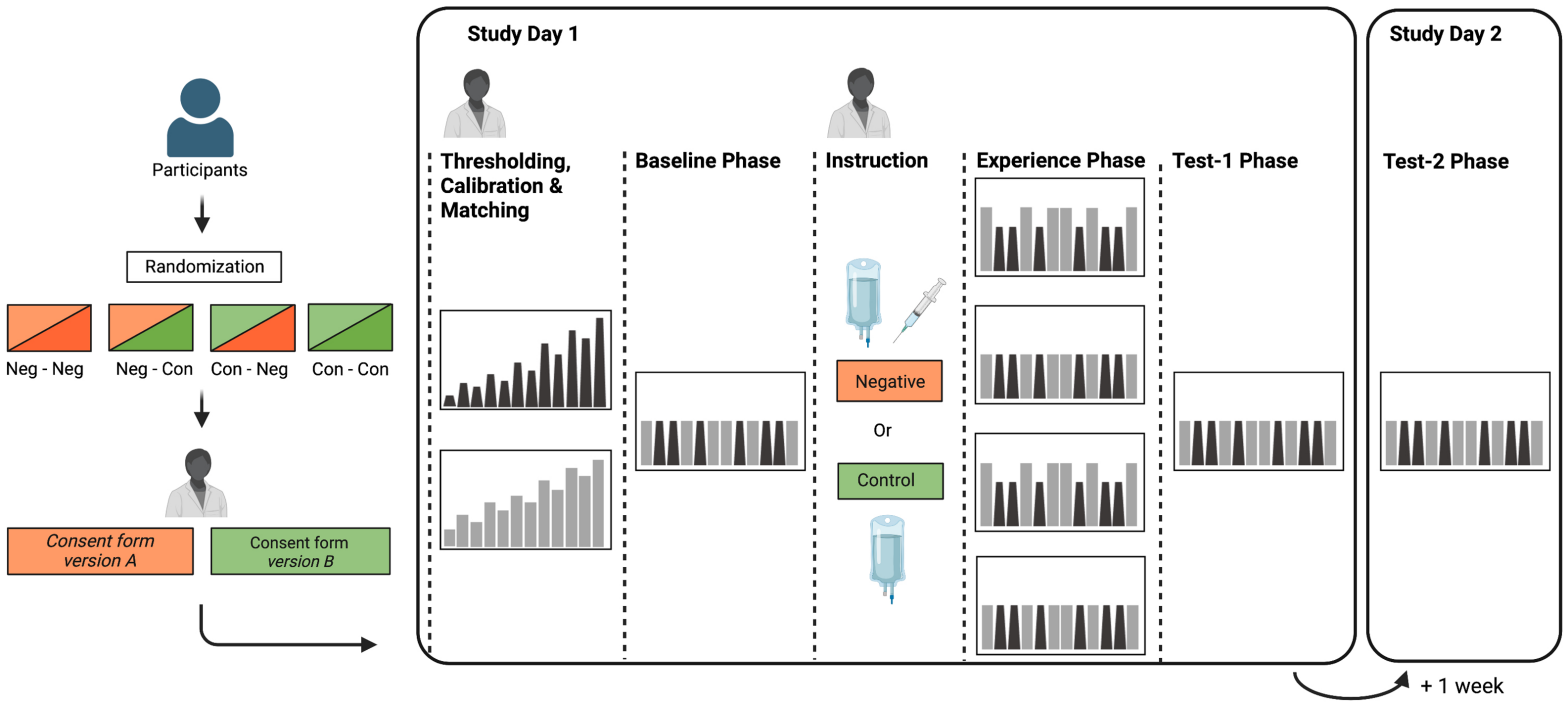
Schematic depiction of the NoVis study design. Participants are initially randomly assigned to one of four groups, combining negative instruction and negative experience (Neg-Neg), negative instruction alone (Neg-Con), negative experience alone (Con-Neg), and a control group without manipulation of negative expectations (Con-Con). On study day 1, all groups undergo an identical thresholding, calibration, and matching procedure and a baseline phase consisting of repeated application of visceral and somatic pain stimuli. Neg-Neg and Neg-Con groups subsequently receive negative treatment instructions regarding the reception of the pain-facilitating drug naloxone, whereas Con-Neg and Con-Con groups are instructed about the application of saline. During the following Experience phase, somatic pain stimuli are surreptitiously increased in Neg-Neg and Con-Neg groups and remain unaltered in Neg-Con and Con-Con. Finally, a test phase identical to baseline is accomplished in all groups. On study day 2 one week later, all groups return and undergo a second test phase consisting of repeated somatic and visceral pain stimulation identical to baseline and Test-1 phases. For thresholding and all experimental phases, gray bars depict somatic pain and black bars represent visceral pain stimuli. The figure was created using BioRender (www.biorender.com).

The study design is visualized in Figure 1, and procedures are described in detail in subsequent sections. The overall idea behind the design is to experimentally create negative treatment expectations through instruction and experience within a treatment context. This is reflected by the key elements of the repeated measures between-subject design containing the between-group factors “treatment instruction” (negative vs. control instruction) and “treatment experience” (negative vs. control experience). To assess the impact of experimental manipulations on outcome measures, subjective and objective responses to a series of visceral and somatic pain stimuli, delivered during several pain stimulation phases, are assessed (repeated factor). Healthy volunteers are randomized into four experimental groups (recruitment goal: *N* = 30 per group). On study day 1, pain thresholding and calibration/matching procedures and a brief pain habituation phase are initially accomplished. This is done to identify individual stimulation intensities for the pain stimulation phases. Several pain stimulation phases are subsequently accomplished (i.e., three on study day 1; one on study day 2), all consisting of a series of individually-calibrated rectal and thermal pain stimuli. After a *Baseline Phase*, all participants receive an i.v. catheter as a salient element of the psychosocial treatment context, with distinct treatment instructions. Briefly, the negative treatment instruction groups are informed that the opioid antagonist naloxone, which amplifies pain, or saline may be administered in a double-blinded manner. Instruction control groups are informed that only saline is administered as part of standardized procedures. In the subsequent *Experience Phase*, thermal pain stimulation intensities are surreptitiously increased in the negative experience groups to create a clearly discernable experience of amplified pain, whereas intensities remain unaltered in the experience control groups. Two *Test Phases* are accomplished, comprising the identical pain stimulation intensities as during *Baseline*. To test short-term effects of expectancy manipulations, the first test phase is accomplished on study day 1 (*Test-1 Phase*). To explore the persistence of effects, another test phase is accomplished 1 week later (*Test-2 Phase*), yet without any of the salient elements of the treatment context (i.e., there is no i.v. and no interaction with the study physician). In all pain stimulation phases, different facets of visceral and somatic pain perception are acquired, along with questionnaires and ratings, psychophysiological recordings, and saliva and blood sampling at various time points (see section 2.5 for details on measures). Note that in order to minimize circadian effects, all experimental testing is accomplished between 13:00 and 17:00 h, with identical starting times for study day 1 and 2 for each participant. Participants are instructed to abstain from physical exercise on the study day, and to omit food and drink (other than water) within 90 min of the scheduled arrival to the laboratory. They are also instructed not to apply body lotion on the abdomen given application of electrodes on the skin.

As primary outcomes, changes in perceived pain intensity and pain unpleasantness will be assessed based on testing for interaction effects and planned group comparisons regarding changes from *Baseline* to the *Test-1 Phase* within each pain modality (study aims 2 and 3). Group differences between pain modalities will be tested based on analysis of delta scores (i.e., changes in visceral > somatic pain responses) (aim 4). Secondary pain-related outcome measures, also tested based on changes from *Baseline* to the *Test-1 Phase* within and between pain modalities, include pain-related expectations (study aim 1), pain recall, and desire to avoid pain. Secondary outcomes related to fear and stress are measured using behavioral, neuroendocrine, and psychophysiological responses, relevant particularly to study aim 6. Responses to pain-predictive cues, i.e., change in cue valence rating and in skin conductance responses, are tested for each pain modality and based on the same experimental phases as for pain-related measures. For neuroendocrine measures, we plan to compare groups with respect to changes from Baseline to subsequent sampling time points. Similarly, group comparisons of differences in heart rate variability and electrogastrogram will be based on analyses of the entire pain stimulation phases (for details, see section 2.4.2). Please note that analyses are planned in two separate steps. Initially, analyses of results of study day 1 will be accomplished. In a second step, analyses comparing data from study day 1 and day 2 (i.e., *Test-1 Phase* vs. *Test-2 Phase*) will be accomplished to explore the possible persistence of nocebo effects over time upon re-exposure to pain stimuli 1 week later (study aim 5). Mediation analyses will be accomplished for aim 1.

### 2.3. Equipment

For the application of phasic visceral pain stimuli, pressure-controlled rectal distensions are delivered using a barostat system (Distender Series II™, 1,300 mL Single Balloon Barostat, G&J Electronics, Toronto, ON, Canada). For the application of phasic somatic pain stimuli, cutaneous thermal stimuli are applied on the skin of the left lower abdomen using a thermode (PATHWAY model CHEPS; Medoc Ltd., Advanced Medical Systems, Ramat Yishai, Israel). For presentation of visual stimuli (i.e., pain-predictive cues) and for digitized online ratings, a commercially available stimulus delivery and experimental control software (Presentation^®^, Neurobehavioral Systems, Albany, CA, United States) is used. For psychophysiological responses, i.e., electrodermal activity (EDA), heart rate variability (HRV), and electrogastrography (EGG) recordings, a commercially available system (BIOPAC MP160 system, Biopac Systems, Inc. Goleta, CA, United States with AcqKnowledge^®^ 5.0.1. software) is used.

### 2.4. Experimental procedures

#### 2.4.1. Thresholding, calibration, and matching of pain stimuli

We have previously established paradigms involving the repeated within-subject application of different aversive stimuli, including visceral pain [e.g., (29, 41, 45)]. These translational “multiple threat paradigms” model the experience of multiple bodily symptoms and offer insight into specificity to the visceral pain modality. To this end, the *a priori* identification of suitable stimulation intensities for each participant, within predefined perceptual intensity ranges and matched across modalities, is a crucial element. This is achieved in a highly standardized, multi-step procedure accomplished prior to the *Baseline Phase*: Initially, individual pain thresholds are determined to serve as anchor for the subsequent calibration and matching procedures. The goal of the calibration is to identify individual stimulus intensities for each pain modality (i.e., a specific pressure for rectal distension-induced pain and a specific temperature for thermal cutaneous pain). Herein, we calibrate a perceptual target intensity of 50 mm perceived pain intensity on VAS with end points labeled “not painful” (0 mm) and “extremely painful” (100 mm), within an acceptable target range of 40–60 mm. Additionally, an intensity of 80 mm on VAS is determined for somatic pain stimuli which is used in the *Experience Phase* for surreptitiously increased thermal pain intensities. The subsequent matching procedure is designed to ensure that perceived pain intensities of visceral and somatic pain stimuli are comparable in terms of perceived pain intensity. To this end, rectal and thermal stimuli are presented simultaneously, and participants are asked to directly compare the intensity of these stimuli using Likert-type response options indicating more, less, or equally painful perception. As long as ratings show a deviation, the intensity of thermal stimuli is successively adjusted until ratings indicate equal perception when compared to visceral stimuli at least twice consecutively. Finally, a brief pain habituation phase is accomplished, comprising presentation of three uncued pain stimuli from each modality, presented in pseudorandomized order and individually rated on VAS for pain intensity, thereby ensuring proper selection of final stimulation intensities. If more than one rating reveals a deviation from either the target range or a divergence of ratings between modalities of >10 mm, stimuli are recalibrated and rematched. Of note, stimulus durations are individually adjusted, aiming at matched durations of ascending and plateau phases of visceral and thermal stimulation, as the inflation and deflation times of the rectal balloon depend on individual pain threshold. For safety reasons, the maximum pressure applied with the rectal balloon is limited to 60 mmHg, and for the thermode a temperature limit is set to 49.5°C in all pain stimulation phases. Furthermore, for ethical considerations, in case of more than two ratings above VAS 90 mm, we refrain from further increasing stimulation intensity and then reduce the intensity if necessary, depending on when such ratings occur.

#### 2.4.2. Pain stimulation phases

The selected objective pain stimulation intensities are applied as follows in the pain stimulation phases: All phases (i.e., *Baseline*, *Test-1*, *Test-2*) are identical, comprising a pseudorandomized series of a total of 12 stimuli (6 visceral, 6 somatic). Of note, the *Experience Phase* is distinctly different in groups randomized to a negative experience which is operationalized by surreptitiously increased thermal pain to the predetermined intensity of VAS 80 mm (see below). Pain stimulus durations are 20 s each, followed by VAS capturing perceptual responses after each stimulus as well as at the end of the series. The order of stimuli is pseudorandomized. To avoid order effects of starting the series with a stimulus from one pain modality, the series starts with either a visceral or a somatic pain stimulus. Additionally, the sequences are programmed to avoid an order containing more than two pain stimuli from the same modality. All pain stimuli are visually cued as a measure of conditioned pain-related fear, and the assignment of a specific cue (i.e., geometric symbol) to a pain modality is counterbalanced. Pain-predictive visual cues appear on a screen 7 s before the pain stimulus, ending at the same time as the pain stimulus (delay conditioning). Total duration of each pain stimulation phase including all digitized ratings is 1,150 s (= 19.17 min). Digital ratings are presented for 12 s, and a fixation cross is shown on the screen in-between stimuli.

#### 2.4.3. Treatment instruction

**Figure 2:**
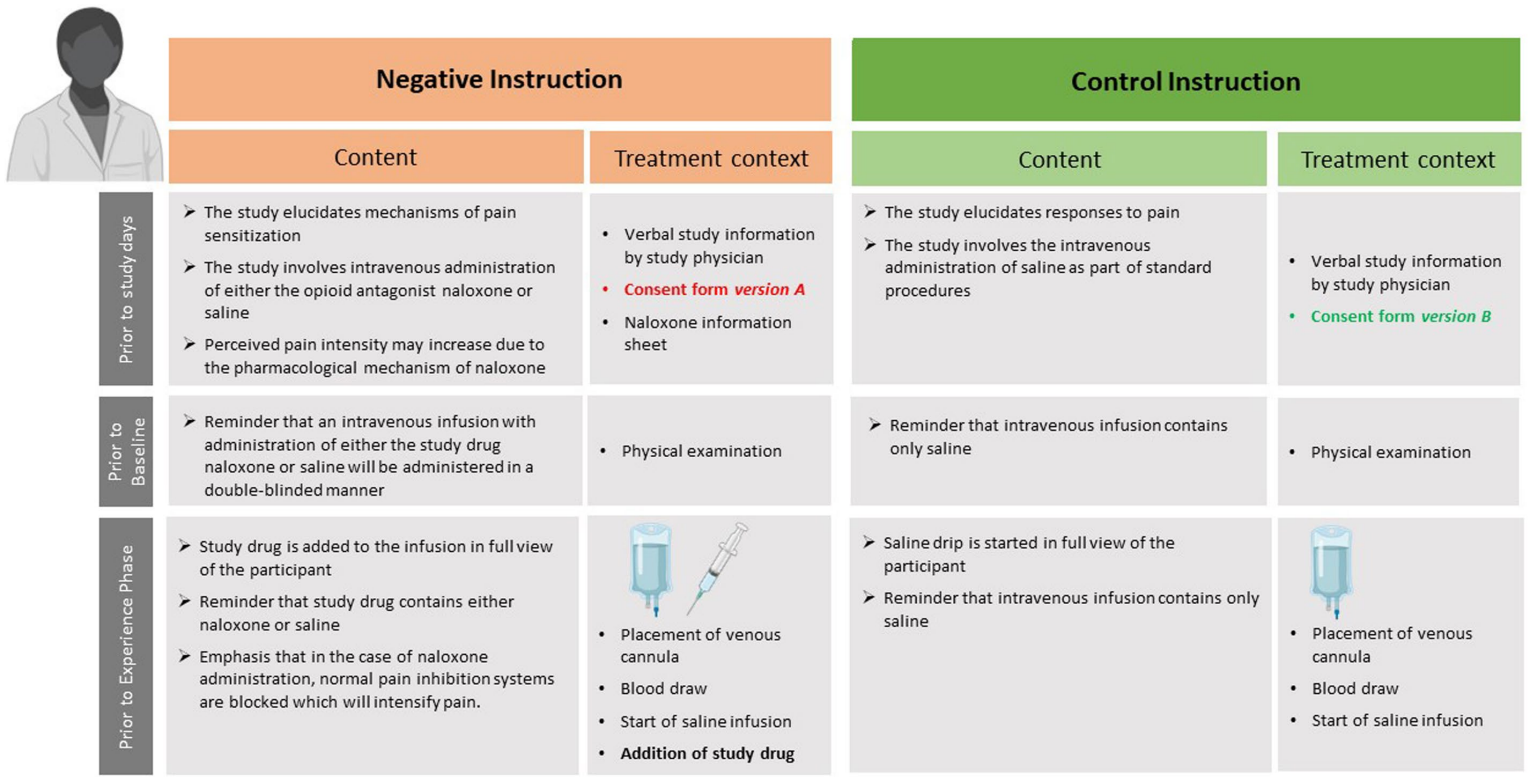
Overview of time point, content, and treatment context of negative or control instructions provided to the respective experimental groups (i.e., Neg-Neg and Neg-Con, compare to Figure 1). The figure was created using BioRender (www.biorender.com).

During the initial recruitment and screening stages, accomplished by study staff prior to randomization, all participants are only informed that the purpose of the study is to evaluate mechanisms of visceral when compared to somatic pain perception. Of note, the general information provided on study-related materials (e.g., study flyers) and during phone screening does not contain any reference to the possibility of receiving a pronociceptive drug or a placebo, but rather focusses on dynamic changes in pain perception upon repeated painful experiences and underlying psychobiological mechanisms. Randomization is accomplished prior to the personal interview with the study physician (for details, see section 2.4.5). Depending on treatment instruction group, participants then receive distinct written and verbal treatment information, and sign distinct versions of consent forms. All treatment-related information is provided by the study physician (author J.L.A.) based on a highly standardized protocol at various time points of procedures, illustrated in Figure 2. For negative instruction groups, the protocol is built on a previously used approach to generate negative expectations about expected pain increase induced by treatment suggestions regarding an intravenous infusion of the opioid antagonist Naloxone (in reality: always saline) [e.g., (40)]. Herein, participants give consent to participating in a placebo-controlled, double-blind study implementing the opioid antagonist Naloxone vs. saline to study pain sensitization and pain responses during an amplified pain experience in two modalities, with a 1:1 randomization ratio (*consent form version A*). Of note, saline is always administered, hence the procedures involve a deception with respect to the randomization ratio and the possibility to receive naloxone (for a critical discussion, please see section 3.2). The briefing contains detailed information about the drug, especially about its pain-enhancing effects, but also about typical clinical uses and known side effects. The “study drug,” which has an official design of the pharmacy of the University Hospital Essen, is placed directly in front of the subject before administration into the i.v. line. In instruction control groups, written and verbal information about the administration of saline is provided (*consent form version B*). The rationale for the study is also adjusted, focusing only on differences between pain modalities. Regarding the i.v. infusion of saline, volunteers are told that it is simply part of standardized procedures. In the context of placing the i.v. line and starting the saline drip, the study physician emphasizes that no active substance is given. Note that perceived treatment allocation is assessed with a questionnaire at the end of study day 2 in all participants and groups. Volunteers randomized to consent form version A, i.e., negative instruction groups, will at the end of the study be fully informed regarding the administration of saline in all participants. The rationale for the deception regarding the possibility of receiving Naloxone will be provided, and participants will be offered the opportunity to withdraw their study data.

#### 2.4.4. Treatment experience

To induce different treatment experiences, in the groups randomized to a negative treatment experience the somatic pain stimulus intensity (i.e., temperature of the thermode) is surreptitiously increased to the pre-calibrated VAS 80 mm target, whereas it remains unchanged in groups randomized to a neutral treatment experience (control groups). Note that rectal distension pressures, preselected to induce moderately painful visceral pain (VAS 50 mm), the objective intensity (i.e., the distension pressure) remains unchanged in all groups. This is done based on conceptual considerations (see section 4, aim 1), including the more precise nature of somatic pain perception and the assumption that generalization effects will occur.

#### 2.4.5. Randomization and blinding

Randomization into one of four groups is accomplished using randomizer.org, stratified by sex. Given that expectancy modulations are at the core of the study, this is accomplished by one dedicated staff member who is not otherwise involved in conducting the study and has no contact with participants beyond initial phone or email contacts and the coordination of the initial appointment/visit with the study physician. The group assignment regarding treatment information group is communicated only to the study physician who explains the study goals and procedures based on version A or B of the consent form and delivers all treatment-related information. As the study physician is therefore necessarily unblinded with respect to the group factor “instruction,” she is blinded regarding the group factor “experience.” This is achieved by a distinct and separate communication between the staff member accomplishing randomization and scientific staff administering pain stimuli, who are therefore unblinded with respect to experience. The study physician is not present during accomplishment of pain phases, and therefore remains blinded throughout. Further, participants’ responses to painful stimuli are not affected by the presence of the study physician. Data entry into a database, data preprocessing, and initial analyses will be accomplished prior to unblinding.

### 2.5. Measures

#### 2.5.1. Psychosocial characteristics

Prior to experimental procedures, participants complete a comprehensive psychosocial questionnaire battery, administered by a central CRC project, acquired with the open-source survey tool LimeSurvey (LimeSurvey GmbH, Hamburg, Germany) (46). Some of these data will not be included for primary analyses of this study but will be used for exploratory investigations and/or as control variables. For the purposes of this study, for characterization of participants as well as for exploratory predictor analyses, we will focus on pain-related cognitions and emotions (Fear of Pain Questionnaire (FOP) (47), Pain Catastrophizing Scale (PCS) (48), Somatosensory Amplification Scale (SSAS) (49), Pain Anxiety Symptom Scale (PASS-20) (50)), and anxiety and stress (Anxiety Sensitivity Index (ASI-3) (51), Perceived Stress Scale (PSS-10) (52), Trier Inventory for Chronic Stress (TICS), short version (53)).

#### 2.5.2. Expectations

Given the key role of negative expectations in the experimental design of the NoVis study, expectations are assessed using three distinct yet complementary measures: (1) The Generic Rating for Treatment pre-Experiences, Treatment Expectations, and Treatment Effects Scale (G-EEE) quantifies treatment expectations and previous treatment experiences (54). Herein, participants complete the scale once in the context of the i.v. infusion on study day 1, and once at the end of study day 2. The G-EEE was specifically developed by members of the CRC 289 as a screening tool for the general assessment and quantification of patients’ treatment expectations and their effects on clinical outcomes. It measures treatment-related expectations as well as prior treatment experiences and current experiences of treatment related effects as potentially relevant predictors of future expectations using uniform scales including improvement as well as worsening and potential side effects. Given the role of nocebo mechanisms in the perception and recall of side effects (55), even in participants randomized to the placebo group of RCT (56), we further administer the Generic Assessment of Side Effects in Clinical Trials (GASE) (57) which provides a more detailed insight into a wide range of bodily symptoms and their severity. (2) VAS ratings of expected pain intensity and expected pain unpleasantness are accomplished for each pain modality at the beginning of each pain stimulation phase, using the same endpoints as for pain-related VAS (see below). To capture conditioned responses to modality-specific predictive cues presented prior to each pain stimulus, changes in cue valence are assessed for each cue once at the beginning and once at the conclusion of each pain stimulation phase. As accomplished in our previous work on conditioning with visceral pain [e.g., (41)], VAS anchors are labeled “very pleasant” (−100 mm) and “very unpleasant” (+100 mm), with the word “neutral” (0 mm) marked in the middle of the VAS. Of note, these cue-related unpleasantness ratings offer the opportunity of parallelized analyses of responses to cues and pain stimuli regarding unpleasantness as a clinically-relevant indicator of emotional valence.

#### 2.5.3. Pain perception and pain recall

As main outcomes, perceived pain intensity and pain unpleasantness are assessed using digitized visual analog scales (VAS) with end points labeled “not painful” (0) and “extremely painful” (100), respectively, for intensity and “not unpleasant (0) and “extremely unpleasant” (100), respectively, for unpleasantness. Of note, these facets of the pain experience are acquired separately for each pain modality, and at several time points: During all pain stimulation phases, perceived pain intensity and unpleasantness are assessed after each painful stimulus, i.e., as trial-by-trial ratings. In addition, at the conclusion of each pain stimulation phase, i.e., after the final pain stimulus, overall perceived intensity, unpleasantness, along with pain-related fear and desire to avoid pain are acquired.

Of note, intensity and unpleasantness constitute two facets of the experience of pain that are interrelated, yet not identical. While perceived intensity primarily reflects sensory-discriminative components of pain perception, pain unpleasantness captures the emotional impact of pain, a dimension that is sometimes overlooked in experimental placebo and nocebo research. This facet of the symptom experience is demonstrably shaped by conditioned pain-related fear (30) and constitutes a key determinant of patient suffering. It may also be particularly sensitive to pain modulation by cognitions and emotions (58), and herein this measure may also be superior given that unlike pain intensity, pain unpleasantness is not experimentally adjusted during calibration, impacting variability.

**Figure 3:**
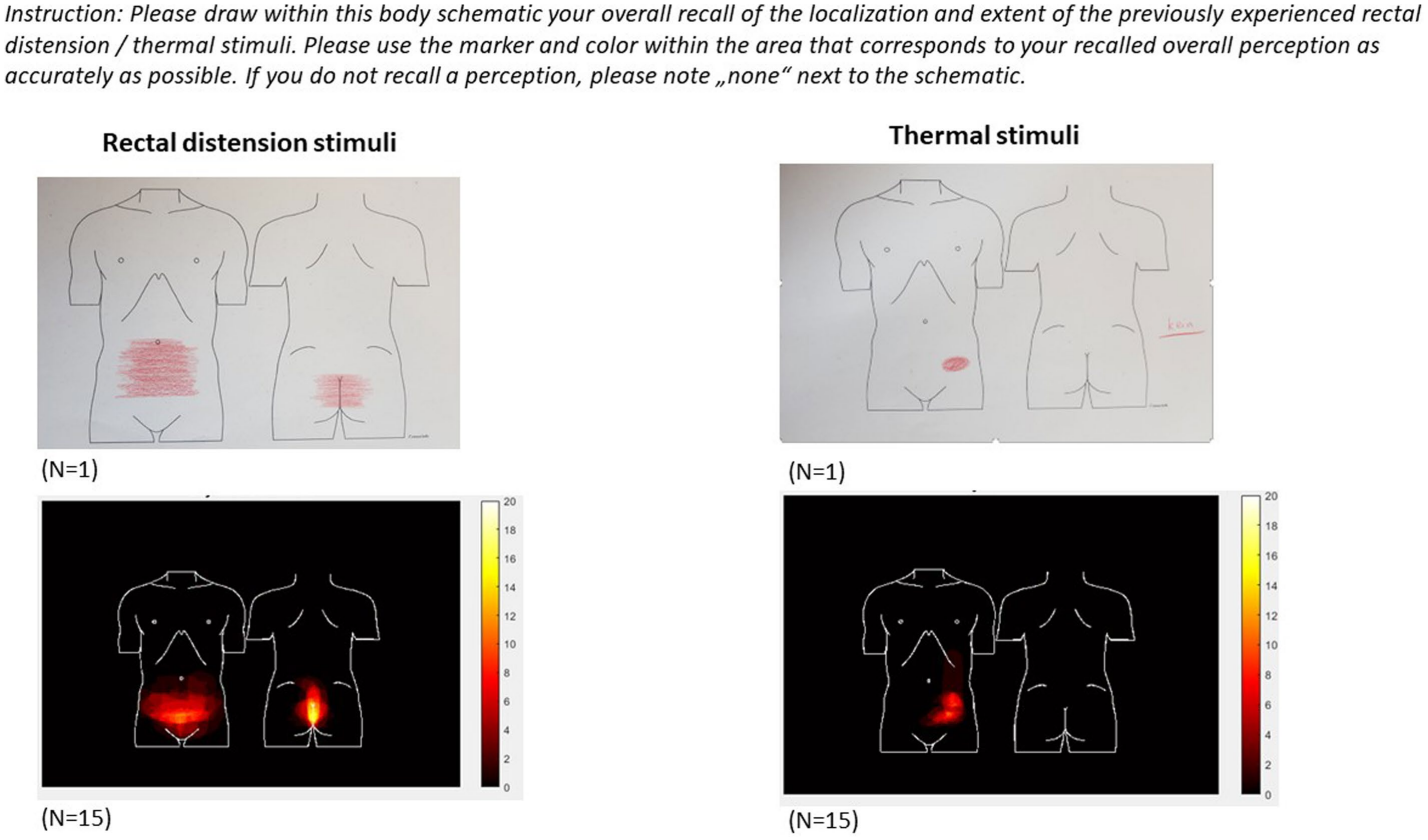
Individual sketches (top) and results from intermediate group analyses (bottom) indicating recalled localization and extent of the body area affected by visceral (left) and somatic (right) pain stimulation experienced on study day 1.

To complement pain-related VAS ratings acquired in immediate (trial-by-trial) or very close temporal proximity (end-of-phase overall ratings), an additional measure is introduced to provide insight into retrospective recall of pain area. To this end, participants indicate their recollection of the overall pain area (i.e., localization and extent) affected by visceral and somatic pain stimuli, respectively, using paper-pencil drawings on a printout of an anatomical sketch of the lower abdominal area and the lower back, respectively, as illustrated in Figure 3. These sketch assessments are accomplished once at the conclusion of the first study day, i.e., after the *Test-1 Phase* and removal of all stimulation and testing electrodes (including removal of the rectal balloon), and once at the beginning of the second study day (i.e., prior to placement of testing equipment), with instruction to remember overall pain extent of the first study day. Analyses will be accomplished as previously described in a study on placebo effects in experimentally-induced esophageal pain (59), rectal pain in IBS (60) and visceral pain modulation (61). Of note, we herein added a sketch visualizing the lower back in addition to a frontal abdominal area sketch to capture differences more fully between pain modalities and increase sensitivity to group differences.

#### 2.5.4. Neuroendocrine and neurochemical variables

As indicators of stress-related activation of the hypothalamus-pituitary–adrenal (HPA) axis and the sympathetic nervous system, respectively, saliva samples are collected for analyses of concentrations of cortisol and alpha-amylase activity. Sampling is accomplished immediately prior to and after each pain stimulation phase on both study days, in parallel to questionnaire assessment of the current emotional state with the State–Trait-Anxiety-Depression-Inventory [STADI, state version (62)]. Additional sampling is accomplished upon arrival on both study days.

Blood draws are accomplished only on study day 1 at three time points (i.e., prior to the *Experience Phase*, after the *Experience Phase*, after the *Test-1 Phase*), enabling later exploratory analyses of ß-endorphin and cholecystokinin concentrations in plasma as putative neurobiological modulators of interindividual variability in pain responses and possibly nocebo hyperalgesia (63–65). To enable analyses of the composition of the microbiome, participants provide a stool sample prior to study day 1.

#### 2.5.5. Psychophysiology

Electrodermal activity (EDA) is continuously recorded during all pain stimulation phases, and skin conductance responses (SCR) to pain-predictive visual cues with a latency of 1 s and until pain onset will be analyzed. The respective peak amplitude during this time interval will be extracted as a proxy of sympathetic nervous system activation reflecting conditioned pain-related fear during pain anticipation, which is a complementary assessment of conditioned responses reflected by analysis of conditioned changes in cue valence (see section 2.5.2) (66). The assessment of conditioned changes in SCR in combination with behavioral responses to conditioned pain-predictive cues builds on our earlier pain-related conditioning studies implementing visceral pain signaled by conditioned visual cues similar to those implemented herein [e.g., (41, 67)].

Heart rate variability analyses will be accomplished over the entire duration of each pain stimulation phase as a measure of stress-related cardiac autonomic regulation (68), based on a continuous one-channel electrocardiogram (ECG) recording. R-peaks of the QRS complexes will be identified to estimate the normal-to-normal (NN) intervals between adjacent QRS complexes resulting from sinus node depolarizations. Standard deviations of NN intervals (SDNN) and the percentage of adjacent NN with a variability of more than 50 ms (pNN50) will be calculated as time-domain parameters reflecting vagally-mediated variations in HRV, along with HF/LF ratio for sympathovagal balance.

Myoelectric activity generated by smooth muscles of the stomach, as can be measured non-invasively with the electrogastrogram (EGG) (69), is reportedly sensitive to stress and emotional arousal in animals and humans (70) and has been shown altered during rectal distension in dogs (71). The EGG has been used in several placebo studies accomplished in the context of nausea [e.g., (72, 73)], or to assess effects of treatment suggestions regarding gastric activity (58, 74–76) in humans, but it has not been applied in the context of acute pain. To explore the EGG as a novel and possibly sensitive measure of nocebo effects relevant to pain, continuous EGG recordings are accomplished in each pain stimulation phase. To acquire data during a phase in which no painful stimuli are implemented, prior to the *Test-2 Phase* on study day 2, a 10-min recording is acquired without any pain stimulation, at the same time serving as habituation phase for possible arousal induced by the experimental set-up. Spectral signatures will be analyzed with respect to power and amplitudes to obtain instability coefficients (IC) reflecting the variability of slow wave activity, the regularity of slow wave activity, the percentage of gastric arrhythmias together with frequency ranges for normal, bradygastric, and tachygastric activity as putative correlates of stress and arousal induced by negative treatment expectations and differences between groups in overall pain responses.

#### 2.5.6. Patient-provider communication

To elucidate facets related to patient-provider relationship and communication, the duration (minutes) of physician-participant communication is recorded, and participants rate perceived warmth and competence of the study physician with 6 items for warmth and competence (77), respectively, at the end of the first study day. Each item is rated on a 5-point scale from 1 (not at all) to 5 (extremely). These ratings capturing the participants’ perspective are complemented by an adapted version of the scale that is completed by the physician who rates the patient/participant on the same dimensions. Items were minimally adjusted for relevant content, and comprise the adjectives intelligent, attentive, anxious, nervous, excited, tense, suspicious, depressed/pessimistic.

#### 2.5.7. Additional measures

In addition to subproject-specific procedures and measures, detailed above, the central projects of the CRC coordinate and administrate standardized assessment of additional measures from all human participants. These include (1) assessment of structural and functional brain connectivity measured at rest in every participant prior to the experimental study protocols, i.e., diffusion tensor imaging (DTI) and resting state functional MRI (rsfMRI) using standardized acquisition and analysis protocols; (2) cortisol awakening response (CAR) and salivary alpha amylase (sAA) activity in all participants as markers of HPA-axis and sympathetic activity, respectively, by obtaining saliva samples on two consecutive days immediately after awaking as well as 30 and 45 min after awakening, and (3) the comprehensive questionnaire battery (mentioned above, see section 2.5.1). These measures will serve analyses across projects aiming to identify predictors of expectancy effects, embedded within the larger CRC approach, along with additional psychological trait and state measures related to stress, negative affectivity, and pain.

## 3. Discussion

### 3.1. Analyses and anticipated results

**Figure 4:**
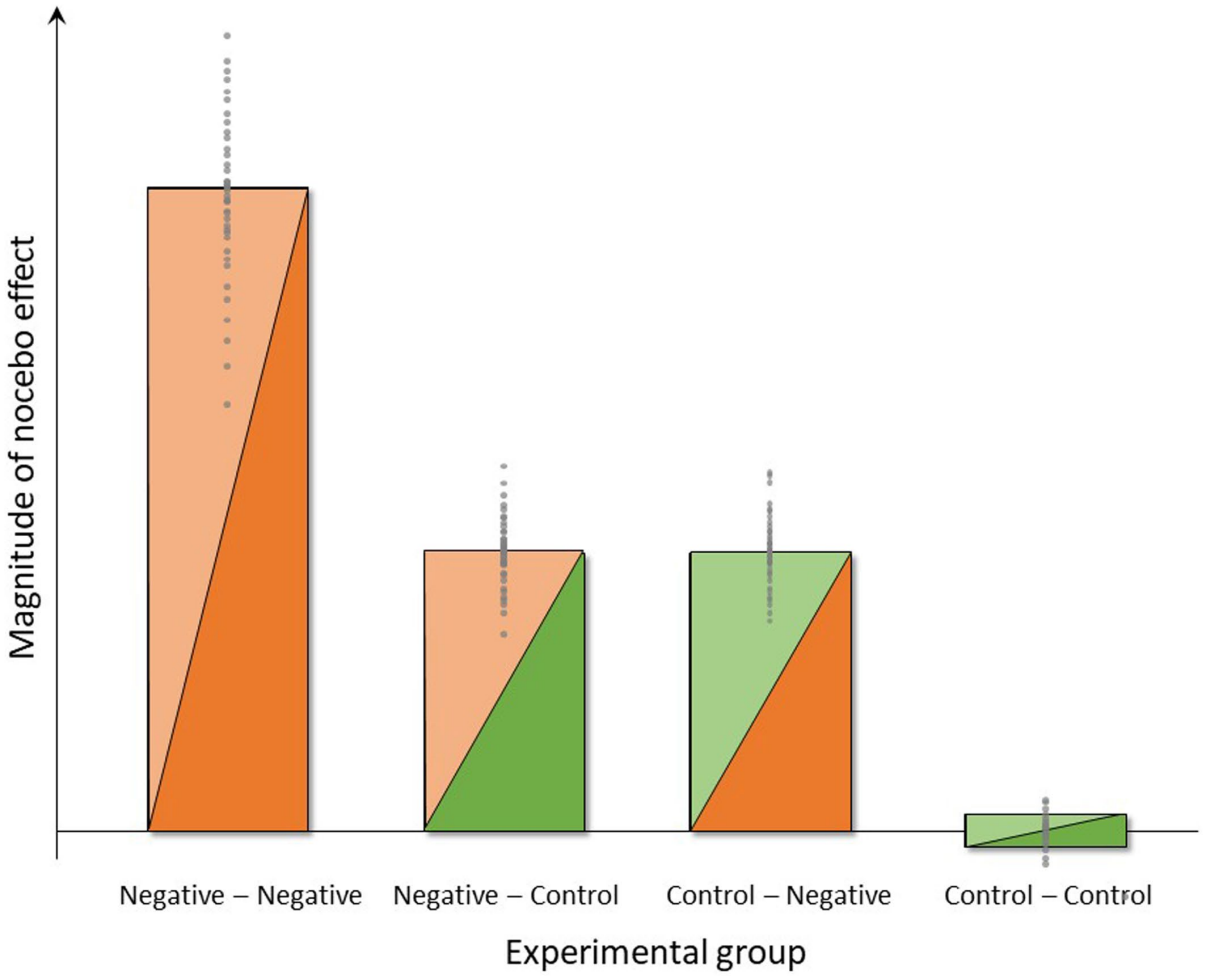
Symbolic depiction of anticipated group differences and interindividual variance in the magnitude of nocebo effects induced by negative instructions and experience (group Neg-Neg), negative instructions and unaltered pain experience (group Neg-Con), control instructions and negative experience (group Con-Neg) or no expectancy manipulations (group Con-Con) in the four experimental groups.

Based on the background and specific aims, we expect the following results based on planned hypotheses-testing as well as on hypothesis-generating analyses of data collected within the NoVis study, analyzed using analysis of variance (ANOVA) and t-tests as primary analysis approach. A symbolic depiction of the expected group differences in the magnitude of nocebo effects in the four experimental groups is provided in Figure 4.

#### 3.1.1. Aim 1: to test dynamic changes in treatment- and pain-related expectations within- and outside of the psychosocial treatment context, i.e., on study days 1 and 2, as mediators of nocebo effects

Given that negative expectations are at the heart of the experimental design of the NoVis study, the first step will be to interrogate group differences in expectations. This will be achieved based on analyses of three distinct yet complementary measures. Firstly, we will analyze G-EEE scores, providing a novel screening tool capturing prior and current treatment-related experiences and expectations, including not only improvement but also worsening as well as potential side effects (G-EEE) (54). As our experimental manipulations are designed to induce negative expectations as mediators of nocebo effects, we anticipate that analyses will confirm the presence of group differences regarding the expected and/or experienced worsening of symptoms. This should be observable in groups that receive negative instruction regarding possible drug-induced pain amplification as well as in groups experiencing pain amplification, with greatest effects hypothesized to occur in the group with negative instruction combined with a negative experience of amplified pain (i.e., the “Negative–Negative” Group).

Secondly, analyses of the G-EEE scores will be complemented and refined by analyses of VAS assessing expected pain intensity and unpleasantness. We assume that expectancy VAS ratings constitute a more sensitive measure as assessments are accomplished in direct timely proximity to pain stimulation phases, allowing us to interrogate dynamic changes in pain-related expectations before and after different experiences of repeated acute pain stimuli during the pain stimulation phases. VAS also directly target pain-related expectations, which are reportedly highly correlated with perceived pain intensity in placebo/nocebo studies [e.g., (40)]. Since pain expectancy ratings are acquired for each pain modality, analyses will allow insight into possible differences in negative expectations for visceral vs. somatic pain stimuli. Therefore, we regard VAS expectancy results as key manipulations checks. It will be particularly interesting to elucidate possible group differences in the experimental groups that were randomized to a negative pain experience (i.e., surreptitiously increased temperature for somatic pain stimuli) when compared to groups in which objective pain stimulation intensities remain unaltered (i.e., experience control groups). We anticipate that compared to control groups, these negative experience groups will demonstrate greater expected pain intensity prior to the *Test-1 Phase* based on the preceding experience of amplified pain intensity. Although we surreptitiously increase only thermal stimulation intensities, we expect to find effects not only for the somatic pain modality but also for the visceral modality. This assumption is based on the hypothesis that a generalization across pain modalities will take place, possibly due to cognitions relating to allocation to drug rather than placebo in groups that were negatively instructed. In other words, we expect that participants who were informed that there exists a 50% chance of receiving a drug that amplifies pain will interpret their perception of greater thermal pain intensity as evidence that they were randomized into the naloxone group, resulting in immediate nocebo effects also for the visceral modality.

Thirdly, learning to anticipate potential bodily harm is essential for adaptive behavior. Via associative learning, conditioned danger cues generate negative expectations along with anticipatory preparatory psychophysiological responses to impending threat (27). Such learned negative expectations can results in hypervigilance and amplification of pain experience, constituting a key mechanism in the translational framework of nocebo effects. We will therefore analyze behavioral and skin conductance responses to conditioned pain-predictive cues as measures of learned conditioned pain-related fear during pain anticipation. We anticipate group differences in the magnitude of conditioned fear responses, with greatest responses expected in the “Negative–Negative” group. Of note, the behavioral read-out we will implement constitutes changes in emotional valence of pain-predictive cues. Emotional valence is relevant to all types of threat, shapes the perception of aversive stimuli, including pain (78), and drives threat-related behaviors like approach and avoidance (79). It is highly relevant to the specificity of visceral pain (30), and sensitive to modulation by placebo/nocebo mechanisms (27, 32). Prior pain-related fear conditioning studies from our own group [reviewed in (27)] and in the broader fear conditioning literature support the notion that conditioned changes in cue valence constitute a sensitive and relevant behavioral measure capturing the formation, as well as the extinction and return of anticipatory fear responses in healthy adults (80) and clinical populations (79). Most recent evidence supports its sensitivity to nocebo mechanisms (81).

#### 3.1.2. Aim 2: to test the effects of negative treatment expectations induced by instruction and/or experience on subjective and objective responses to acute visceral and somatic pain stimuli

Regarding perceived pain intensity and unpleasantness as main outcomes, we expect that both negative instruction as well as negative experience will result in hyperalgesia for both pain modalities, i.e., greater pain intensity and unpleasantness ratings during the *Test-1 Phase* vs. *Baseline* in experimental groups “Control-Negative” and “Negative-Control” when compared to the group “Control–Control.” Of note, the bars depicting expected results (Figure 4) indicate a similar magnitude of nocebo effect for the groups “Control-Negative” and “Negative-Control.” In other words, we assume in this visualization that the efficacy or impact of instruction and experience will be comparable. It should be stressed, however, that there is no prior evidence testing such an assumption in similar experimental approaches, and it will be interesting to reveal whether the impact of instruction vs. experience is indeed comparable as a basis for translation into practice. If, for example, the impact of negative experience is more “powerful” in terms of inducing sustained nocebo effects when compared to instruction, this will inform strategic and conceptual developments aiming to minimize nocebo effects in clinical practice. Further, we anticipate that the negative experience of objectively increased thermal pain will impact on the perceptual response also to visceral pain (e.g., via generalization and/or cognitive attribution effects, as explained above), exerting an impact also on both pain modalities during the *Test-1 Phase*. Regarding objective measures related to pain, as secondary outcomes, we will explore group differences in psychophysiological responses expecting greater sympathetic activation reflected by differences in HRV and EGG measures in the “Control-Negative” and “Negative-Control” when compared to the group “Control–Control.”

#### 3.1.3. Aim 3: to determine if a negative treatment experience enhances the magnitude of negative instruction effects

This study aim constitutes the most crucial and arguably most clinically relevant aspect of this study with respect to both novelty and putative implications. In clinical reality, negative prior experiences with the same or a similar treatment (context) are very common, especially in patients with chronic symptoms that are difficult to treat, as in DGBI. Therefore, experimentally testing not only the effects of instruction and experience separately but rather their combination is important since the combination of instruction and experience has been shown to be particularly effective in eliciting a nocebo effect (39). We therefore herein anticipate an interaction of these factors, resulting in greatest nocebo effects induced by the combination of negative instruction and negative experience, i.e., in the “Negative–Negative” group, as visualized in Figure 4. The magnitude of the increase in nocebo effect in this group over the “Control-Negative” and “Negative-Control” groups will depend on effect sizes in those groups, which cannot be estimated based on existing data. It is however conceivable that the additional increase will be substantial in case additive or even synergistic effects occur. If this were the case, clinical application of nocebo research would have to target the role of (prior) treatment failure within the context of patient-provider communication in order to avoid or minimize nocebo effects. Given prior evidence of transfer or carry-over effects of prior treatment history to the response to a novel treatment (82), including evidence that the effects of treatment failure generalize across different routes of drug administration (83), the clinical relevance of treatment history (i.e., treatment experience) cannot be underestimated. At the same time, it is also possible that only the combination of instruction and experience will result in a discernable nocebo effect, possibly resulting from small or even absent negative expectations resulting from our experimental manipulations when accomplished separately. It is also conceivable that the study setting involving pain stimuli already contains nocebo mechanisms that impact on the “Control–Control” group as well. The information that painful stimuli from two pain modalities will be delivered, which is obviously given to all experimental groups, may constitute a driver of negative expectations that our additional experimental manipulations must override for us to be able to detect effects. The magnitude of such effects cannot be estimated without additional control groups, especially a group that does not receive any painful stimuli. Hence, it should be noted that the symbolic depiction of the expected magnitude of nocebo effects in Figure 4 as essentially zero in the “Control–Control” group, reflects the concept of the study design rather than the true absence of any negative expectancy effects in this group expecting and experiencing aversive painful stimuli.

#### 3.1.4. Aim 4: to compare the magnitude of nocebo effects in the visceral versus the somatic pain modality

The present study is the first to allow insight into nocebo effects across modalities, aiming to test the notion that effects of negative expectations may be enhanced for the visceral when compared to the somatic modality. This is based on earlier findings supporting a distinct role of fear and stress for perceptual responses to pain arising from the viscera and partially distinct underlying neural circuitry (29, 41). It is therefore intriguing to assume that interoceptive, visceral perceptions may be more modifiable by psychological factors, including negative expectations. Putting this to a test within this comprehensive study, we expect greater and more sustained nocebo effects on visceral vs. somatic pain responses. We anticipate that this will be particularly evident in measures reflecting emotional-motivational facets of pain, which we herein assess with VAS of perceived pain unpleasantness and visceral pain-related fear in response to conditioned predictive cues.

#### 3.1.5. Aim 5: to assess the persistence of nocebo effects on responses evoked by a re-exposure to pain stimuli 1 week later

We expect sustained effects of expectancy manipulations accomplished on study day 1, which we interrogate based on group comparisons of the *Test-2 Phase*, as well as based on analyses of change scores from the *Test-1* Phase to the *Test-2 Phase*. We anticipate that groups with negative instructions and/or negative experience will expect greater pain on study day 2, i.e., prior to the *Test-2 Phase*, even though elements of the treatment context, such as an i.v. line, will not be present on that day. If we indeed can show such effects, this would be an important finding toward explaining how nocebo effects may be maintained in clinical reality over time and repeated symptom experiences. Given our considerations about differences between pain modalities, especially the putatively greater psychological modulation of visceral pain, we expect sustained effects to be enhanced for the visceral pain modality. We will focus on elucidating modality-specific facets of pain recall, aiming to further elucidate the notion of a memory bias for visceral pain, expanding on our previous work (84) and the notion that visceral pain-related fear memories may be particularly resistant to extinction (41).

#### 3.1.6. Aim 6: to identify predictors of interindividual variability in the magnitude of nocebo effects and to elucidate associations with state and trait variables relevant to stress, anxiety, and fear at the behavioral, psychophysiological and neuroendocrine levels

We expect that negative expectation effects are at least in part mediated or moderated by state and trait characteristics reflective of stress, anxiety or fear, and arousal, and will explore this via group comparisons and exploratory correlational and regression analyses. A role of trait or state negative emotions or stress in nocebo effects has been proposed based on experimental evidence that, however, almost exclusively came from research in somatic pain models [reviewed in (27)]. Briefly, studies have suggested a role of dispositional fear and anxiety as well as state anxiety and stress. There also exists first evidence that HPA-axis and sympathetic mediators may play a role in nocebo responses. In visceral pain, existing evidence is inconclusive, with more findings failing to support a role of stress and anxiety than those supporting their contribution, unless stress is experimentally induced or augmented (40). It is also conceivable that the role of stress or stress mediators in the generation and maintenance of nocebo effects has not been consistently detected due to the absence of longer-term or repeated testing, essentially omitting knowledge about memory. Stress and stress mediators demonstrably impact on associative learning and memory processes (84), including conditioned pain-related learning and extinction, which are distinctly altered in patients with IBS (28, 85). Stress may facilitate a reactivation of the pain-related memory trace, exerting an impact on pain-related outcomes that emerge during re-exposure to painful stimuli, herein on study day 2. Given these considerations, the present study assesses a variety of measures reflecting different facets of stress, anxiety, and fear on psychological and neurobiological state and trait levels, which will allow for hypothesis-generating exploratory analyses for hopefully more firm conclusions.

### 3.2. Strengths and limitations

The study “NoVis” has strengths as well as limitations. Strengths include the experimental study design that systematically manipulates negative expectations via instruction and/or experience, thereby testing effects of treatment information via suggestion and effects of learning/conditioning as the two principle psychological mechanisms underlying expectancy effects not only separately, but also in combination. As such, this is the first study addressing the interaction of factors that are known to induce nocebo effects from a translational perspective. This will not only fill gaps in knowledge from a mechanistic perspective, but has also the potential to be of great value for clinical application in terms of preventing or reducing nocebo effects in the treatment of DGBI and other visceral pain conditions. Similar advantages arise from the implementation of interoceptive visceral and exteroceptive somatic pain stimuli within a translational “multiple-threat paradigm.” These paradigms more closely model the experience of multiple symptoms arising from the viscera and other bodily sites, reflecting patients’ clinical reality of experiencing aversive intestinal and extraintestinal symptoms, either as part of the typical clinical phenotypes, such as in inflammatory bowel diseases, or as part of frequent comorbidities between chronic pain conditions. For mechanistic research into psychological mechanisms relevant to understanding the normal and adaptive specificity of pain responses for the visceral modality, these paradigms have proven to be highly instrumental not only in healthy individuals [e.g., (22, 29)] but also in patients (86). Indeed, when repeatedly confronted with visceral and somatic painful stimuli matched to intensity, healthy volunteers perceive visceral stimuli as more unpleasant, fear-evoking, and threatening. Distinct differences are also observable at the level of neural representations of visceral versus somatic pain [e.g., (41, 86)], in line with a proposed greater biological salience of visceral signals which may shape perception and pain-related cognitive and emotional responses, including learning and memory processes underlying nocebo effects. Another strength is the 2-day study paradigm with a re-exposure to the same pain stimuli 7 days after first testing without a treatment, allowing to elucidate longer-term effects and crucial aspects of learning and memory processes, including explicit memories (i.e., reported pain recall and extent of recalled pain area) as well as implicit effects of prior experience (i.e., responses to conditioned cues; expectations regarding pain intensity). The sustainability of nocebo effects over time, especially the impact of a previous negative treatment experience on the response to subsequent exposure to contextual or sensory stimuli cannot be underestimated in terms of their clinical relevance, as illustrated by carry-over effects demonstrated for the somatic pain modality (82, 83). Finally, the combination of subjective and objective measures to capture diverse facets relevant to pain, fear, and stress will allow not only hypothesis-testing but also hypothesis-generating analyses that can broaden the horizon of possible mediators and moderators and that have the potential to further conceptual and methodological advances.

Limitations and potential pitfalls are equally important to consider. Firstly, while all participants are truthfully informed that the purpose of the study is to elucidate psychobiological mechanisms underlying changes in visceral compared to somatic pain perception upon repeated painful experiences, the study procedures of consent form A (i.e., negative instruction groups) involve a deception. To induce negative treatment-related expectations, participants are informed that they have a 50% chance of receiving either a pronociceptive drug or saline, mimicking clinical treatment scenarios characterized by uncertainty regarding symptom worsening or the occurrence of side effects or adverse events, such as in clinical trials. In reality, herein the probability of receiving saline is 100%. The administration of saline and the scientific justification for providing different instructions to experimental groups will be disclosed after the conclusion of the study, at which time participants will have the opportunity to withdraw their data. While a comprehensive discussion of ethical considerations is beyond the scope herein, the use of deception is not unequivocal, requires justification, and rightfully constitutes an issue of critical debate. There exist different solutions, such as open-label placebos [e.g., (87)], with limited applicability for nocebo research, and authorized consent [e.g., (88)]. A comprehensive work focused on ethical considerations specifically pertaining to nocebo effects is provided herein (89). Secondly, while interindividual variability is a highly relevant aspect to consider, it could at the same time hamper the detection of small effects. The sample size with a goal of *N* = 30 per experimental group was determined based on a prior power calculation and is consistent with (or even larger than) similar experimental work on visceral pain modulation [e.g., (40)] and the broader field [reviewed in (90)]. While the inclusion of diverse and complementary secondary outcome measures at different levels (behavioral, psychophysiological, brain) is consistent with sustainability goals in scientific research and will allow exploratory analyses of diverse study aims, multiple tests and comparisons will be accomplished. This comprehensive study protocol provides readers with maximal transparency, however, there is a risk of potential inflation of *p*-values and false positive results, and findings will have to be interpreted with due caution, requiring replication in independent samples and/or settings. Thirdly, normal changes in perceptual responses to repeated visceral vs. somatic stimuli may differ between modalities as well as between individuals, as suggested by our previous work in similar paradigms. Careful calibration and matching accomplished herein to overcome this challenge may not fully prevent this, with a habituation to cutaneous thermal pain stimuli over time constituting the most likely pitfall. This will be quantifiable in the “Control–Control” group, and of course statistically-controlled for, but analyses directly focusing on differences between pain modalities induced by expectancy manipulations will nevertheless have to overcome this variability. As we cannot with certainty exclude that spontaneous changes in perception occurring independent of our expectancy manipulations interfere with their impact in a modality-specific manner, we will conduct initial analyses within each pain modality separately, i.e., without including pain modality as a repeated factor. Dedicated analyses will be accomplished to elucidate differences between modalities, e.g., using delta scores on the modality difference, as previously accomplished [e.g., (41)]. Finally, limitations arise from the fact that the study is conducted in a tightly-screened sample of relatively young healthy adults. We will recruit men and women, offering opportunities to explore effects of sex/gender, especially within predictor analyses (aim 6). Regarding planned group analyses (e.g., for aims 2–4), (sub)sample sizes are likely too small for any firm conclusions regarding effects of sex/gender, yet exploratory analyses can be accomplished and are interesting given that females and males may differ with respect to the generation of expectancies by suggestions and conditioning processes, respectively (91). Mitigating possible gender-related confounding remains a challenge in experimental research, especially in studies involving visceral pain stimuli posing feasibility restrictions for larger samples that would provide adequate statistical power for detecting sex differences. We and others have previously accomplished some dedicated research on this important subject in the context of visceral pain and visceral pain modulation [e.g., (31, 92–94), as recently reviewed in (9)]. Overall, volunteers herein will not be representative of diverse age, cultural, and ethnic groups, and certainly not of vulnerable individuals or even patients with DGBI and multiple comorbidities. As a complex psychosocial phenomenon, expectancy effects in psychosocial treatment contexts are modulated by societal, cultural, and numerous individual factors, which we will only partially be able to capture and interrogate herein. This is particularly relevant for analyses aiming to address the putative contribution of trait- and state measures relevant to stress and anxiety. While it is tempting to speculate that interactions between stress and negative expectancy effects — whether induced by suggestions or conditioning—could be increased in patients, the translation from healthy individuals tested in experimental laboratory settings to clinical populations is far from unequivocal. Clearly, it is a challenging yet indispensable future research task to elucidate mediators and moderators of placebo and nocebo responses in patients experiencing chronic visceral pain in order to delineate the putative role of stress in nocebo effects in laboratory research, everyday clinical practice as well as in clinical trials.

### 3.3. Implications and outlook

In conclusion, research on the nocebo effect has begun to unravel the functional and neurobiological mechanisms underlying visceral hyperalgesia and hypervigilance. Emerging research from the pain field and beyond supports the crucial role of negative expectations in shaping clinical outcomes. Further, nocebo effects—or rather nocebo-relevant mechanisms—are relevant beyond their treatment implications. They may indeed also play a role in the transition from acute to chronic pain as well as in the maintenance and perpetuation of symptom chronicity, consistent with a biopsychosocial disease model of chronic pain. At the same time, remaining gaps in knowledge call for more mechanistic research in order to integrate and clarify the contribution of different mediators and moderators, offering fascinating future directions in this rapidly evolving field. Only a more refined understanding of nocebo mechanisms will ultimately allow the development and testing of effective ways to reduce these effects in clinical settings. Therefore, the scientific and clinical potential of elucidating negative expectation effects on different pain modalities is enormous, and this study has the potential to provide a sound basis for translational research into application in the treatment of patients with DGBI and beyond.

## Author contributions

JA: Conceptualization, Data curation, Investigation, Methodology, Writing – original draft, Writing – review & editing. AI: Conceptualization, Methodology, Supervision, Writing – original draft, Writing – review & editing. SE: Conceptualization, Funding acquisition, Project administration, Supervision, Writing – original draft, Writing – review & editing.

## Footnotes

^1^https://treatment-expectation.de/en/
